# Can two multimodal psychomotor exercise programs improve attention, affordance perception, and balance in community dwellings at risk of falling? A randomized controlled trial

**DOI:** 10.1186/s12889-022-13725-5

**Published:** 2022-07-11

**Authors:** Hugo Rosado, Jorge Bravo, Armando Raimundo, Joana Carvalho, Gabriela Almeida, Catarina Pereira

**Affiliations:** 1 Departamento de Desporto e Saúde, Escola de Saúde e Desenvolvimento Humano, Universidade de Évora, Largo dos Colegiais 2, Évora, Portugal; 2 Comprehensive Health Research Centre (CHRC), Universidade de Évora, Évora, Portugal; 3 Faculdade de Desporto, Universidade do Porto, Porto, Portugal; 4 CIAFEL - Research Centre in Physical Activity, Health and Leisure, Porto, Portugal

**Keywords:** Older adults, Falls, Psychomotor intervention, Whole-body vibration, Exercise therapy and rehabilitation, Action boundary

## Abstract

**Background:**

Falls are associated with cognitive and physical function deterioration. Attention decline, inaccurate affordance perception, and balance impairment are considered to be risk factors for falls. Furthermore, few studies have reported psychomotor intervention as a fall prevention program. This study aimed to investigate the effects of two multimodal programs on attention, perceptual and stepping-forward boundaries, and balance in community-dwelling older adults at risk of falling.

**Methods:**

Fifty-one community-dwelling older adults were recruited to participate in a 24-week randomized controlled trial. Participants (75.4 ± 5.6 years) were randomly assigned to one of three groups: the 1) multimodal psychomotor program [EG1], 2) combined program (multimodal psychomotor program + whole-body vibration program) [EG2], and 3) control group. Participants were assessed at baseline, at post-intervention, and after a 12-week no-intervention follow-up period.

**Results:**

The within-group comparisons showed significant improvements in attention and balance in EG1 and EG2 after the intervention (*p* <  0.05). The magnitudes of the treatment effects were similar in both EGs, ranging from medium to large. Decreases in the fall rate were also observed in EG1 (− 44.2%) and EG2 (− 63.0%) (*p* <  0.05). During the follow-up period, these improvements in attention were maintained, while those in balance were reversed in both EGs. No significant differences between groups were found.

**Conclusions:**

These study results suggest that both multimodal exercise programs were effective for fall prevention and were well tolerated by the participants. Specifically, EG1 and EG2 showed identical improvements in attention, and EG2 presented a slightly larger enhancement in balance and a larger decrease in the fall rate. Our findings demonstrate the benefits of maintaining the psychomotor intervention program by itself or in combination with the whole-body vibration program to prevent cognitive and physical function deterioration.

**Trial registration:**

ClinicalTrials.gov Identifier:NCT03446352. Date of registration: February 26, 2018.

## Background

According to the United Nations, the number of older adults aged 65 years or over is increasing faster than all other age groups [1]. Following this trend, the aging process is related to an increase in falls, such that one-third of community-dwelling older adults aged 65 years or more, experience at least one fall each year, resulting in substantial economic costs [2]. This evidence highlights the importance of developing effective strategies and programs to prevent fall occurrences and manage fall risk factors to maintain independence and quality of life [2, 3].

Related to the aging process, a link has been established between cognitive decline and fall risk since cognitive function and motor maintenance share restricted neural resources [4]. Within cognitive function abilities, attention is a specific element of executive functions (EF) [5]. Evidence from neuroimaging studies focusing on structural or physiological changes (e.g., cerebral white matter and brain volume) suggests that a decline in EF is related to an increased fall risk [5, 6]. According to O’Halloran et al. [7], brain changes promote a larger variability in sustained attention, which is strongly associated with fall risks. Additionally, the selective attention described as a fundamental EF has also been related to falls [6].

Similarly, age-associated locomotor skills deterioration can lead to inaccurate perceived action limits, whereby it is essential to recognize the respective action boundary (e.g., perceptual and stepping-forward boundary), especially in community-dwelling older adults [8]. Accordingly, affordances, that is, possibilities for action, are a concept involving the relationship between the action possibilities of the individual (e.g., maximum stepping-forward length) under a particular set in an environment [9, 10]. However, recent literature has shown that older adults frequently overestimate their motor abilities, specifically their action boundary as a step length [8]. This is particularly relevant and especially true for fallers because those who overestimate their step length reveal more signs of motor deterioration, which can lead to an increase in fall risk [8, 11]. Moreover, perceptual overestimation can also potentially induce balance impairment and consequent falls [11]. Despite the previous findings, no experimental studies on fall prevention programs were found focusing on affordance perception, particularly the perceived and real action boundary, enhancing the need for further investigations.

Additionally, balance impairment is related to falls and is one of the most often used and recommended components for integration into fall prevention programs as well as one of the most effective at reducing the rate and risk of falling, especially when incorporated into multimodal exercise programs [12].

The body and brain adapt in response to consistent cognitive and physical stimuli [4]. In this line, previous studies have proposed the concept of neuroplasticity over aging [13], with the possibility for older people to improve their performance through single or combined cognitive-motor intervention programs. Nevertheless, the potential improvements in fall prevention programs depend on the type of tasks and training proposed [3, 12]. Single cognitive training programs such as computer-based cognitive training can positively induce improvements in motor control, specifically in locomotor coordination, reducing fall risk [4]. Likewise, exercise alone (e.g., balance training and functional exercises) is also considered effective at reducing the rate of falls (23%) and the number of fallers (15%) [12]. However, the current literature suggests that a combined intervention focusing on cognitive and motor exercise challenges may promote additional benefits [14, 15]. Despite this, few studies concerning this cognitive-motor interactive training on risk factors for falls have been carried out [16], highlighting the need for further investigations, particularly on community-dwelling older adults.

In this line, evidence supports the use of psychomotor interventions focusing on the body and movement as a means for expression to enhance the cognitive, motor, and relational aspects of psychomotor aging [17]. Specifically, a psychomotor intervention may induce improvements in the age-related deterioration of the previous processes [13]. However, there is a lack of studies focusing on psychomotor intervention as a fall prevention program [18]. Likewise, whole-body vibration (WBV) has been shown to be effective in improving balance in older adults through neurophysiological mechanisms (i.e., the mechanical vibration conducted to the body, in association with the respective biological effects), reducing the risk and incidence of falls [19, 20]. This method may also lead to an enhancement of EF [21]. However, to our knowledge, an intervention program that combines both methods has not yet been studied, particularly on fall prevention programs. Thus, the objective of this study was to investigate the effects of two multimodal programs on attention, perceptual and stepping-forward boundaries, and balance in community-dwelling older adults at risk of falling.

## Methods

### Trial design

The present study was designed as a 24-week randomized controlled trial (RCT), single-blinded, with a three-arm parallel assignment. Community-dwelling older adults from Évora (Portugal) were allocated into three groups (allocation ratio 1:1:1): experimental group 1 (EG1) was assigned a multimodal psychomotor program; experimental group 2 (EG2) was assigned a combined program (multimodal psychomotor program + WBV); and the control group (CG) was asked to maintain their daily life activities. After the study finished, those in the CG were offered an identical fall prevention program. This trial was conducted between March 2018 and January 2019, and it was previously registered at ClinicalTrials.gov (NCT03446352). Also, this study was reported in accordance with the CONSORT guidelines for RCTs (http://www.consort-statement.org).

### Participants

Participants were male and female community-dwelling older adults recruited in community settings as the local senior university and recreational centers via pamphlets. In each community setting, verbal communication was used to present our study and for answers to any possible doubts. The older adults who expressed interest to participate were scheduled for the baseline evaluation.

A minimum sample size of 45 participants was required (15 participants per group) to detect a treatment difference, calculated by the online G*Power software, under the following parameters: α = 0.05 and power = 0.95. Accounting for an expected dropout rate of 20%, a minimum of 60 participants were recruited for this study.

The inclusion criteria comprised the following: a) age ≥ 65 years old; b) classified with moderate or high physical independence according to the Composite Physical Function (CPF) scale (≥ 18 points) [22]; c) participants who had experienced at least one fall in the previous 6 months or were identified with a high risk of falling according to the result in the Fullerton Advanced Balance (FAB) scale (≤ 25 points) [23]. Exclusion criteria comprised: a) the presence of cognitive impairment (≤ 22 points in the Mini-Mental State Examination - MMSE) [24]; b) walking dependently (e.g., with mobility aids); c) musculoskeletal, cardiovascular, and neurological conditions [25]; and d) attending physical and/or cognitive structured exercise programs preceding 6 months [26].

**Fig. 1:**
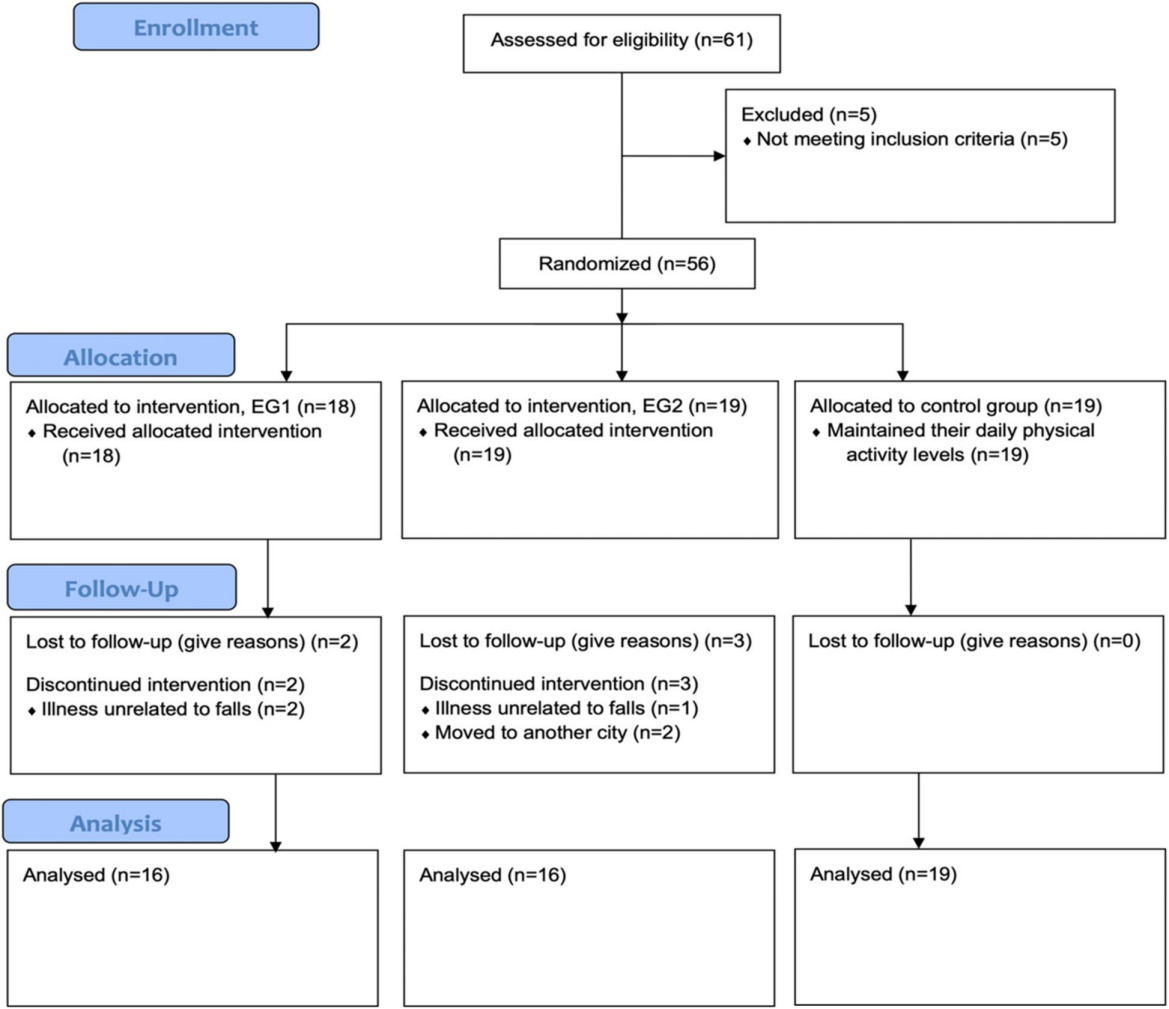
Flow Diagram

Initially, sixty-one older adults were assessed for eligibility and agreed to participate in the study as described in Fig. 1. Five participants did not fulfill the inclusion criteria, which remained a total of fifty-six participants (47 women and 9 men). For participants who were enrolled in this study, simple randomization was performed according to the “Random Team Generator” (https://www.randomlists.com/team-generator) into EG1 (*n* = 18), EG2 (*n* = 19), and CG (n = 19). An investigator with no clinical involvement in the trial performed the randomization.

All the study participants were volunteers and gave their written informed consent. This study was approved by the University of Évora Ethics Committee - Health and Well Being (reference number 16012) and conducted in accordance with the Declaration of Helsinki.

### Procedures

A trained evaluator in the rehabilitation sciences field individually assessed all participants at baseline, at post-intervention (24 weeks), and after a 12-week no-intervention follow-up. The evaluator was blinded to participants’ allocation. Cognitive and other measures assessed by questionnaires were performed in a laboratory silent room. Affordance perception, physical function and body composition assessments were performed in a laboratory hall. All assessments were preceded by the protocoled explanation and/or demonstration performed by the evaluator.

Data collection was performed at the University of Évora laboratories.

### Outcome measures

#### Attention

Selective and sustained attention was assessed by the d2 Test of Attention, which was demonstrated to be a valid and reliable measurement in older people [27]. Participants had 20 s in each of the 14 lines of the test to identify and mark the letter “d” with two dashes (above or below the letter), as quickly as possible. Measures of performance comprised items processed (n); items recognized correctly (n); total efficacy (n), which indicates the relationship between speed and thoroughness in the task; concentration index (n), which reflects the ability to concentrate; fluctuation rate (n), which indicates the consistency in the task execution; and percentage of errors (%).

#### Affordance perception

The perceptual and stepping-forward boundary was assessed by the stepping-forward affordance perception test, established as a valid, accurate, and reliable tool for fall risk assessment in community-dwelling older adults [8]. The estimated stepping-forward and real stepping-forward distances were collected as described by Almeida et al. [8]. In addition, the absolute error (|real-estimated distances|) and the error tendency measuring the magnitude and direction error (overestimation: real < estimated distances; or underestimation: real > estimated distances), (over- or underestimation) were also computed.

#### Balance

Multidimensional balance was assessed by the FAB scale, which is considered a valid and reliable instrument designed to assess independently living older adults. This scale comprises 10 individual tests, such that each one ranged from 0 (worst) to 4 points (best), and the “Total FAB scale” (sum of the test scores) ranged from 0 (worst) to 40 points (best) [23].

#### Falls

The occurrence of falls, respective circumstances (e.g., type/place of fall), and consequent injuries were assessed by means of an interview following a 13-item script, although only the occurrence of falls was used in this manuscript. A fall was defined in accordance with the definition proposed by the World Health Organization “as *an event which results in a person coming to rest inadvertently on the ground or floor or other lower level*” [28]. The number of fall occurrences in the previous 6 months was recorded retrospectively at baseline and at post-intervention.

#### Secondary outcomes measures

Each session exercise intensity was assessed by the Borg Rating of Perceived Exertion scale, ranging from “very, very light” (6 points) to “very, very hard” (20 points), measured [29]. Satisfaction level achieved through each exercise session was assessed by the Caregiver Treatment Satisfaction (CTS) questionnaire, ranging from “extremely dissatisfied” (1 point) to “extremely satisfied” (5 points) [30]. Cognitive performance was assessed by the MMSE [24]. Sociodemographic characteristics were assessed through an interview based on a script. Body mass index was calculated by dividing weight by height squared (kg/m^2^), in which the participant’s height (m) was measured shoeless in a stadiometer (Seca 206, Hamburg, Germany), and the weight (kg) was measured using an electronic scale (Seca 760, Hamburg, Germany). Physical independence was assessed by the CPF scale, ranging from 0 (worst) to 24 points (best) [22]. Based on the previous 12-item CPF scale score, participants were classified as low functioning (< 18 points), moderate functioning (18–23 points), and high functioning (24 points). Finally, physical activity (the sum of walking, moderated and vigorous physical activity) was assessed by the International Physical Activity Questionnaire (IPAQ) using the metabolic equivalent of task ([MET]-min/week), calculated as activity duration*frequency per week*MET intensity [31].

### Multimodal exercise programs

Participants randomly engaged in one of the two EGs (3x/week on non-consecutive days; 75 min/session). Each EG was divided into two classes, without differences, with up to 10 participants. A master’s degree therapist in psychomotor therapy planned and directed both EG sessions under the supervision of a university Sports Sciences professor. The therapist who planned and operationalized both exercise intervention programs did not participate in the assessments. When the EGs participants were absent for 3 consecutive sessions, the missed sessions were rescheduled to maintain the established attendance level (≥ 80%). EG1 assigned a multimodal psychomotor program, with 75-minute sessions, that privileged the body and movement as mediators. This program integrated simultaneous neurocognitive (focusing on executive function training) and motor (focusing on physical fitness performance) stimulation through several exercises designed to promote general physiological and specific neurophysiological stress in the involved mechanisms. EG2 assigned a combined program (multimodal psychomotor program + WBV program; starting with 72 + 3 min/session and ending with 69 + 6 min/session, respectively). The time allocated to the WBV program was proportionally withdrawn from each phase of the multimodal psychomotor program. Regarding the WBV program performance, the participants stood shoeless on the side-alternating vibratory platform (Galileo® Med35) in a semi-squat position. The exercise time ranging from 45 to 60 (s), the number of series ranging from 4 to 6, and the frequency ranging from 12.6 to 15 Hz progressively increased over intervention. The amplitude (3 mm) and resting time between series (60 s) remained equal throughout the intervention.

The complexity and intensity of both programs increased with sessions (planned for moderate intensity: until approximately 13 points at RPE scale). Each session was divided into 5 phases: beginning ritual (~ 5 min), warm-up (~ 10 min), main section (~ 50 min), cool down (~ 5 min), and a finishing ritual (~ 5 min). After a neuromuscular activation warm-up, the main section was implemented, comprising multimodal exercises. In this phase, neurocognitive-, motor-, and sensorial-specific exercises promoting simultaneous cognitive (e.g., attention - to assign different commands to different actions), perceptual (e.g., motor planning - to imagine geometric figures on the floor and then execute the movement), and motor (e.g., balance - body sport balance disc and fitball exercises to change the base of support) stimulation were performed on identical alternated periods (10–15 min). During the cool-down, relaxation techniques and exercises involving body awareness/scheme were performed. Finally, at the finishing ritual, participants recorded intensity and satisfaction levels through the RPE scale and CTS questionnaire, respectively.

### Data analysis

To ensure participant confidentiality and anonymity, a code was attributed to each participant. Data were analyzed using the SPSS software (v. 24.0, IBM SPSS Inc.). The significance level for all the statistical analyses was established at *p* <  0.05.

Descriptive data are expressed in terms of the mean and standard deviation (SD) for quantitative variables and frequency (%) for categorical variables. Differences (∆) between each evaluation moment (baseline, post-intervention, and follow-up) were calculated for all variables by the formula *∆ = moment*_*x*_ *- moment*_*x-1*_, and the proportional changes were computed such as *∆% = [(moment*_*x*_ *- moment*_*x-1*_*)/moment*_*x-1*_*] × 100).*

The Kolmogorov-Smirnov test and Levene homogeneity of variances test were used to evaluate the normality of the data distribution. Since much of the data were not normally distributed, non-parametric tests were performed, namely, the Friedman test for comparisons within groups followed by the related pairwise post hoc test and the Kruskal-Wallis test for comparisons between groups followed by the independent pairwise post hoc test. In the case of two related samples, the Wilcoxon test was carried out for within-group comparisons. Additionally, to perform comparisons regarding qualitative variables (error tendency variables), Cochran’s Q test was used for within-group comparisons, and the chi-squared test was used for between-group comparisons.

The magnitude of the treatment effect was determined following the instructions for non-parametric tests [32] and according to Cohen’s method, in which the effect size (ES) was computed as r *=* (Z/√N). Standardized classification for small (0.10), medium (0.30), and large (0.50) effects was used [33].

## Results

Table 1 provides the participants’ characteristics at baseline and no significant differences between groups were found.

**Table 1:** Participant’s characteristics at baseline

	EG1 Prevalence or Mean ± SD	EG2 Prevalence or Mean ± SD	CG Prevalence or Mean ± SD	***P***-value
Age (years)	74.3 ± 5.4	74.7 ± 5.5	76.8 ± 5.8	0.407
Sex, female (%)	14 (87.5)	15 (93.8)	13 (68.4)	0.124
Educational level (years)	6.0 ± 2.6	6.1 ± 3.4	7.0 ± 5.3	0.997
Cognitive performance (points)	27.7 ± 1.7	28.2 ± 1.7	28.5 ± 1.6	0.332
Body mass index (kg/m^2^)	29.1 ± 3.0	28.6 ± 4.3	28.1 ± 4.4	0.648
Physical independence (points)	21.5 ± 2.7	20.8 ± 2.2	21.5 ± 2.8	0.554
Physical activity (MET-min/week)	927.0 ± 557.9	953.4 ± 638.5	740.4 ± 520.9	0.611
Number of falls within the last six months (n)	1.13 ± 0.8	1.19 ± 1.0	1.11 ± 0.3	0.993

*SD* standard deviation, *EG1* experimental group attending the multimodal psychomotor program (*n* = 16), *EG2* experimental group attending the combined program: multimodal psychomotor program + WBV (*n* = 16), *GC* control group (*n* = 19). Significant differences between groups, *p* <  0.05

A total of fifty-one participants completed this RCT study. Those who dropped out of the study (*n* = 5) had similar characteristics compared to participants who completed the multimodal exercise programs. Regarding the attendance sessions, both EGs met the established attendance level, with similar results on the 75 sessions (EG1: 82.3% vs. EG2: 84.3%). Regarding the tolerability and satisfaction level of the multimodal exercise programs, both EGs had identical results, as shown by the RPE scale (EG1: 12.9 ± 0.4 vs. EG2: 13.2 ± 0.3) and CTS questionnaire (EG1: 4.98 ± 0.3 vs. EG2: 4.99 ± 0.1), respectively.

Table 2 presents the results for cognitive function, namely, selective and sustained variables. At baseline, all groups presented similar results, and no statistically significant differences were found between groups in cognitive variables. On post-intervention evaluation and on follow-up evaluation, between-group comparison did not detect significant differences between the three study groups in these variables.

**Table 2:** Impact of the multimodal exercise programs in selective and sustained attention variables

	Baseline (A) (Mean ± SD)	Post-intervention (B) (Mean ± SD)	Follow-up (C) (Mean ± SD)	***P***-value^**a**^	Pairwise Comparison
Selective and sustained attention					
Items processed (n)					
EG1	254.8 ± 68.0	292.3 ± 86.9	288.8 ± 82.9	0.009	A < B, C
EG2	265.1 ± 78.2	303.3 ± 82.8	300.4 ± 91.5	0.001	A < B, C
CG	244.8 ± 75.5	250.0 ± 81.2	249.9 ± 78.1	0.854	–
*P*-value^b^	0.855	0.204	0.210		
Items recognized correctly (n)					
EG1	96.6 ± 27.2	110.0 ± 37.3	108.9 ± 35.5	0.047	–
EG2	101.8 ± 36.2	120.4 ± 33.9	118.8 ± 36.9	< 0.001	A < B, C
CG	95.5 ± 34.8	95.0 ± 39.8	99.8 ± 36.2	0.076	–
*P*-value^b^	0.893	0.160	0.295		
Total efficacy (n)					
EG1	236.3 ± 70.3	276.9 ± 90.5	272.2 ± 85.7	0.003	A < B, C
EG2	251.2 ± 81.2	292.2 ± 84.3	286.4 ± 92.1	< 0.001	A < B, C
CG	227.3 ± 73.9	228.6 ± 84.9	234.3 ± 80.2	0.076	–
*P*-value^b^	0.801	0.136	0.312		
Concentration index (n)					
EG1	90.0 ± 32.1	105.9 ± 40.6	103.0 ± 38.0	0.002	A < B, C
EG2	97.8 ± 38.2	116.3 ± 36.3	115.8 ± 39.3	< 0.001	A < B, C
CG	89.9 ± 38.1	88.3 ± 44.7	96.1 ± 39.1	0.141	–
*P*-value^b^	0.858	0.143	0.293		
Fluctuation rate (n)					
EG1	11.1 ± 2.6	12.6 ± 3.3	11.1 ± 3.5	0.207	–
EG2	12.7 ± 6.0	10.3 ± 2.6	10.4 ± 3.1	0.637	–
CG	12.9 ± 4.9	11.8 ± 4.6	10.2 ± 3.2	0.047	–
*P*-value^b^	0.262	0.182	0.575		
Percentage of errors (%)					
EG1	7.8 ± 6.9	6.2 ± 5.6	6.6 ± 5.5	0.895	–
EG2	6.0 ± 5.3	4.2 ± 3.8	5.3 ± 5.8	0.611	–
CG	7.7 ± 6.0	10.4 ± 9.3	7.6 ± 7.3	0.141	–
*P*-value^b^	0.549	0.068	0.423		

*SD* standard deviation, *EG1* experimental group attending the multimodal psychomotor program (*n* = 16), *EG2* experimental group attending the combined program: multimodal psychomotor program + WBV (*n* = 16), *CG* control group (*n* = 19)

^a^within-group comparisons

^b^between-group comparisons

<: significant differences within groups, *p* < 0.05

The within-group comparisons showed significant improvements between the baseline and post-intervention evaluations in both EGs, particularly in the variables “Items processed”, “Items recognized correctly”, “Total efficacy”, and “Concentration index”. Specifically, both EGs increased the total number of items processed in the variable “Items processed” (∆% EG1: 14.7%, *p* = 0.014; ∆% EG2: 14.4%, *p* = 0.006), improved the efficacy of performing the task in the variable “Total efficacy” (∆% EG1: 17.2%, *p* = 0.006; ∆% EG2: 16.3%, *p* = 0.001), and increased the concentration in the variable “Concentration index” (∆% EG1: 17.8%, *p* = 0.003; ∆% EG2: 19.0%, *p* = 0.001). Significant improvements were also found by correctly identifying more “d” letters with 2 dashes in the variable “Items recognized correctly” (∆% EG2: 18.3%, *p* = 0.001). Similarly, the post hoc test pairwise comparisons revealed significant differences in the same variable, “Items recognized correctly” (∆% EG1: 13.9%, *p* = 0.022). Furthermore, significant improvements between the baseline and the follow-up evaluations were found in the variables described above, namely, in “Items processed” (∆% EG1: 13.4%, *p* = 0.040; ∆% EG2: 13.3%, *p* = 0.003), in “Items recognized correctly” (∆% EG2: 16.8%, *p* = 0.001), in “Total efficacy” (∆% EG1: 15.2%, *p* = 0.018; ∆% EG2: 14.0%, *p* = 0.002), and in “Concentration index” (∆% EG1: 14.4%, *p* = 0.031; ∆% EG2: 18.5%, *p* = 0.002). In addition, the post hoc test pairwise comparisons showed significant differences in the variable “Fluctuation rate” in the CG (∆%: − 20.8%, *p* = 0.043). Regarding the ES within-groups between the baseline and the post-intervention evaluations, from the previous variables, it ranged from 0.47 (medium) to 0.54 (large), in EG1, and from 0.48 (medium) to 0.51 (large), in EG2, while between the baseline and the follow-up evaluation ranged from 0.43 (medium) to 0.52 (large), in EG1 and was medium (0.48), in EG2.

Table 3 shows the results for the affordance perception and physical function - multidimensional balance - variables. At baseline, all groups presented similar results, and no statistically significant differences were found between groups on the perceptual and stepping-forward boundary variables or on multidimensional balance. On post-intervention evaluation and on follow-up evaluation, between-group comparison did not detect significant differences between the three study groups in these variables.

**Table 3:** Impact of the multimodal exercise programs in the affordance perception and balance variables

		Baseline (A) Prevalence or Mean ± SD	Post-intervention (B) Prevalence or Mean ± SD	Follow-up (C) Prevalence or Mean ± SD	***P***-value^**a**^	Pairwise Comparison
Perceptual and stepping-forward boundary						
Estimated stepping-forward (cm)						
	EG1	53.1 ± 10.4	53.5 ± 14.0	54.1 ± 12.1	0.779	–
	EG2	56.3 ± 12.6	58.4 ± 9.5	60.2 ± 13.3	0.051	–
	CG	61.3 ± 13.5	58.5 ± 11.8	55.6 ± 13.4	0.340	–
	*P*-value^b^	0.069	0.178	0.454		
Real stepping-forward (cm)						
	EG1	60.6 ± 17.8	64.8 ± 15.3	62.9 ± 14.0	0.156	–
	EG2	65.7 ± 10.9	67.3 ± 11.7	66.5 ± 15.0	0.432	–
	CG	69.5 ± 16.5	64.4 ± 19.4	61.7 ± 18.4	0.157	–
	*P*-value^b^	0.339	0.878	0.734		
Absolute Error (cm)						
	EG1	10.4 ± 8.0	11.4 ± 8.5	11.0 ± 8.7	0.528	–
	EG2	9.4 ± 6.5	10.3 ± 6.0	9.2 ± 7.1	0.939	–
	CG	8.8 ± 7.5	9.9 ± 7.1	10.0 ± 6.4	0.555	–
	*P*-value^b^	0.644	0.928	0.852		
Error tendency (%)						
Overestimation	EG1	12.5	6.3	31.3	0.039	–
Underestimation		87.5	93.8	68.8		–
Overestimation	EG2	0	12.5	31.3	0.042	–
Underestimation		100	87.5	68.8		–
Overestimation	CG	15.8	31.6	31.6	0.276	–
Underestimation		84.2	68.4	68.4		–
	*P*-value^b^	0.199	0.199	0.223		
Multidimensional balance (points)						
	EG1	27.1 ± 4.9	31.5 ± 3.7	29.0 ± 4.7	< 0.001	B > A, C
	EG2	27.6 ± 5.1	32.4 ± 4.1	29.9 ± 4.9	< 0.001	B > A, C
	CG	29.7 ± 3.2	29.5 ± 3.7	28.9 ± 3.5	0.351	–
	*P*-value^b^	0.248	0.054	0.751		

*SD* standard deviation, *EG1* experimental group attending the multimodal psychomotor program (*n* = 16), *EG2* experimental group attending the combined program: multimodal psychomotor program + WBV (*n* = 16), *CG* control group (*n* = 19)

^a^within-group comparisons

^b^between-group comparisons

<: significant differences within groups, *p* < 0.05

As seen in Table 3, the within-group comparison showed no significant differences between the three evaluation data on perceptual and stepping-forward boundary variables, except in the variable “Error tendency”. Cochran’s Q test revealed significant differences in the variable “Error tendency” in both EGs at the follow-up evaluation, in which an increase in the number of participants overestimating the perceived stepping-forward boundary was observed.

The within-group multidimensional balance variable comparison showed significant improvements between baseline and post-intervention in both EGs. As shown in Table 3, after the 24-week intervention, EG1 improved by approximately 4.4 more points (∆% = 16.2%, *p* <  0.001). Similar results in the same variable had EG2, which improved by approximately 4.8 more points (∆% = 17.4%, *p* <  0.001). Additionally, differences between the post-intervention and follow-up evaluations were also observed in this variable, in which both EGs showed a worse score in the follow-up evaluation than in the post-intervention evaluation (∆% EG1: − 7.9%, *p* = 0.018; ∆% EG2: − 7.7%, *p* = 0.011). The respective ES between the baseline and post-intervention evaluations was large in EG1 (r: 0.60) and EG2 (r: 0.62). Between the post-intervention and follow-up evaluations, the ES was also large (r: 0.59) in both EGs, representing a considerable decrease in performance.

Last, concerning the number of falls, at baseline, all groups presented similar results, and no statistically significant differences were found between groups in the number of falls. The within-group comparison analysis indicated significant improvements by reducing the number of falls between the baseline and post-intervention evaluations (fall number EG1: 1.13 ± 0.8 vs. 0.63 ± 0.7, *p* = 0.021; fall number EG2: 1.19 ± 1.0 vs. 0.44 ± 0.7, *p* = 0.008). In turn, no differences were observed in the CG (1.11 ± 0.3 vs. 0.95 ± 1.0, *p* = 0.405).

## Discussion

The present study aimed to investigate the effects of two multimodal exercise programs on attention, affordance perception, and balance in community-dwelling older adults at risk of falling. First, both the multimodal psychomotor program and the combined program (multimodal psychomotor program + WBV program) were demonstrated to be effective for fall prevention and were well tolerated. Second, results suggested that both programs induced significant improvements in cognitive and physical risk factors for falls, particularly in regards to attention and multidimensional balance, with similar treatment effect magnitudes. These results complement recent literature knowledge suggesting that combined programs may potentialize the benefits of interventions designed for older adults [15], particularly in regards to risk factors for falls [14, 16]. In particular, our findings showed similar improvements in attention in both EGs and a slightly larger enhancement in balance in EG2. The improvements found in the present study were also observed concerning the number of falls, with a significant decrease in the fall rate in EG1 and especially in EG2, which showed a larger decrease. Furthermore, after a 12-week no-intervention follow-up period, these improvements in both EGs were maintained in attention and were reversed on balance.

The adherence rate in our EGs study (83.3%) was in line with other fall prevention programs [12]. In the same way, the EGs participants in the present study reported similar levels on the Borg Rating of Perceived Exertion scale (moderate intensity) compared to those reported in a previous study [34]. Likewise, the satisfaction level shown in EG1 (4.98 ± 0.3) and EG2 (4.99 ± 0.1) in the current study was identical to the results reported in Linde and Alfermann [34].

For cognitive function, the within-group comparisons showed that both multimodal exercise programs induced improvements in selective and sustained attention variables, with an ES ranging from medium to large. Few studies have used the d2 Test of Attention in community-dwelling older adults. In this line, the 16-week study of Linde and Alferman [34] showed improvements in the concentration index in the physical, cognitive, and combined (physical + cognitive) groups compared to the CG. However, the cognitive group had a larger ES (0.88) than the combined (0.64) or physical (0.51) groups. Although few studies have shown cognitive benefits of a WBV program [21], no additional benefits were found in EG2. The improvements in attention variables in our multimodal exercise programs could be explained by the fact that combined interventions promoting simultaneous dual task activities (cognitive + motor tasks) may promote additional benefits [14], and could provide changes in the prefrontal cortex, which is considered an area age-sensitive to changes in several cognitive domains such as attention [35]. Also, combined interventions could lead to a reduction in attention demand [36]. The findings at the 12-week follow-up period of the present study are consistent with the 12-week follow-up study of Jehu, Paquet and Lajoie [36], in which the physical and combined (physical + cognitive) groups improved EF, reducing the attention demand, and sustained these enhancements at the follow-up. Additionally, our study findings showed that the variable “total efficacy” was the only variable that remained with a large ES within EG1. However, contrary to the 12-week follow-up study of Linde and Alfermann [34], in which only the physical group retained improvements in the concentration index, our study’s EGs maintained their results in selective and sustained attention.

Concerning the affordance perception variables, the within-group comparisons at the follow-up evaluation showed an increase in the overestimation values (error tendency) only in the EGs. Starting with the error tendency results, at baseline, all groups underestimated more the perception-action ability, especially EG2, which could work as a protective mechanism for falls [8]. However, 12 weeks of detraining was sufficient for a decrease in the perception-action ability, inducing a significant increase in the overestimation values. The fact that the participants performed the stepping-forward affordance perception test in a controlled environment, with no potential risk of falling and more confidence, may also have influenced the results. Given the lack of experimental studies on these matters, step length overestimation in older adults has been reported in other cross-sectional studies [11]. Caffier et al. [11] reported significant differences in the step length estimation error (overestimation) in older adults with and without a risk of falling. Given the importance of an accurate perception-action ability, especially in an overestimated performance, future studies should incorporate exercises focusing on anticipatory motor planning. The rationale for this recommendation is based on the fact that older adults prepared an action with a larger anticipation to achieve the same accuracy than younger groups, in addition to greater prefrontal cortical activation [37]. Likewise, possible recommendations for future investigations include a larger long-term fall prevention program (e.g., 12 months) focusing even more on affordances and perception-action ability and motor imagery training. In this line, a recent systematic review suggested that the use of motor imagery training, which appeals to the imagination of an action without the respective motor execution, may improve risk factors for falls, such as balance and mobility in older adults [38].

For physical function, both multimodal exercise programs induced improvements in multidimensional balance, with a large ES. Although both EGs presented a similar ES, the combined exercise program presented a slightly larger ES. Few studies have reported the effects of a WBV program in addition to an exercise program in community-dwelling older adults. The present study findings are in line with the 8-week study of Pollock et al. [39], although the setting was designed for frail older adults. In the Pollock et al. study [39], the addition of a WBV program to balance and strength training resulted in similar enhancements in balance in both groups (exercise alone vs. exercise + WBV). A recent 4-week study also detected significant improvements in balance in a combined program (WBV + unstable shoes) compared to a CG that received WBV with standard shoes [40]. These improvements in balance were found in both groups at post-intervention for the FAB scale score (combined program: 30.7 vs 35.2 points; CG: 31.9 vs. 35.6 points) and were maintained after a 4-week follow-up, only in the combined program (35.2 vs. 35.1 points) [40]. Contrary to the follow-up results of previous studies, in which the balance results remained unchanged after a 4-week follow-up [40] or a 24-week follow-up [39], the improvements in balance in the present study were no longer evident in both EGs after 12 weeks of detraining. The ES in both EGs remained large, revealing a decrease in performance.

Regarding the number of falls, both EGs showed a decrease in the fall rate post-intervention (EG1: -44.2%; EG2: − 63.0%). Although no significant differences were found between groups, the combined exercise program induced a higher decrease in the fall rate. In agreement with the results of the present study, cognitive-motor interference training has been demonstrated to be effective for preventing falls in older adults [16]. As mentioned before, few studies have focused on psychomotor intervention as a fall prevention program. The fall rate in the psychomotor intervention group of the Freiberger et al. study [18] was observed at the 12-month follow-up, and no significant reduction in falls was found. In addition, the improvements in cognitive and physical risk factors for falls in EG2 in our study and the neurophysiological mechanisms induced by WBV training may have promoted additional benefits in the fall rate. In fact, WBV training as a single intervention can lead to a reduction in fall incidence in 12-week intervention programs [41]. However, the low frequency applied by the WBV program in the current study is in line with other studies [41, 42]. In addition, higher-frequency vibration training (> 40 Hz) can lead to reduced immediate neuromuscular performance [41].

The present study has strengths and limitations. The strengths include an RCT design with a long-term intervention comprising two multimodal exercise programs barely studied in fall prevention programs. To the best of our knowledge, this is also the first RCT focusing on the perceptual and stepping-forward boundary as a risk factor for falls in community-dwelling older adults. Current limitations include a single-blinded design and the dropout rate in the EGs (9.8%), although the present study showed a smaller dropout rate than other studies [34], and the remaining sample fulfilled the minimal sample size defined by the G*Power software. Even so, the decrease in sample size may have limited the statistical power of the study. Although descriptive data related to between-group comparisons were not contradictory to within comparisons, no significant differences were found as regards inferential comparisons between groups, namely at post-intervention. Despite the predominance of women in the present study (82.4%), these values are in line with other studies that reported approximately 80% of women in their survey [40]. Last, it would have been interesting to assess measures such as the Activities-specific Balance Confidence Scale to evaluate other parameters that could influence the outcomes and recorded the number of falls at the follow-up evaluation.

## Conclusions

The results of this RCT study suggest that the multimodal psychomotor program and the combined program (multimodal psychomotor program + WBV program) were effective and well tolerated in community-dwelling older adults at risk of falling. Both multimodal exercise programs induced improvements in risk factors for falls, particularly in attention and balance, with similar treatment effect magnitudes, ranging from medium to large in EG1 and EG2. Specifically, both EGs revealed identical improvements in attention, and the combined program presented a slightly larger enhancement in balance. Additionally, both EGs showed a decrease in the fall rate post-intervention, especially the combined program. After 12 weeks of detraining, the positive effects evidenced in both EGs were sustained in attention but reversed in balance. Our findings advocate the benefits of maintaining the multimodal psychomotor program as a single or combined intervention with WBV to prevent cognitive and physical function decline. Furthermore, given the increase in the aging trend, this study reveals two promising approaches to use as a fall prevention program in community-dwelling older adults, which can reduce the expensive health and social and economic costs from falls.

## Data Availability

The datasets used and/or analyzed during the current study are available from the corresponding author upon reasonable request.

## Abbreviations

EF: Executive function
WBV: Whole-body vibration
RCT: Randomized controlled trial
EG1: Experimental group 1
EG2: Experimental group 2
CG: Control group
CPF: Composite Physical Function
FAB: Fullerton Advanced Balance
MMSE: Mini-Mental State Examination
RPE: Borg Rating of Perceived Exertion
CTS: Caregiver Treatment Satisfaction
IPAQ: International Physical Activity Questionnaire
MET: Metabolic equivalent of task
SPSS: Statistical Package for the Social Sciences
SD: Standard deviation
ES: Effect size
